# Effects of A6E Mutation on Protein Expression and Supramolecular Assembly of Yeast Asparagine Synthetase

**DOI:** 10.3390/biology10040294

**Published:** 2021-04-03

**Authors:** Thunyarat Surasiang, Chalongrat Noree

**Affiliations:** Institute of Molecular Biosciences, Mahidol University, Salaya, Nakhon Pathom 73170, Thailand; thunyarat.sur@student.mahidol.edu

**Keywords:** asparagine synthetase, mutation, protein expression, assembly, yeast

## Abstract

**Simple Summary:**

Certain mutations causing extremely low abundance of asparagine synthetase (the enzyme responsible for producing asparagine, one of the amino acids required for normal growth and development) have been identified in humans with neurological problems and small head and brain size. Currently, yeast is becoming more popular in modeling many human diseases. In this study, we incorporate a mutation, associated with human asparagine synthetase deficiency, into the yeast asparagine synthetase gene to demonstrate that this mutation can also show similar effects as those observed in humans, leading to very low abundance of yeast asparagine synthetase and slower yeast growth rate. This suggests that our yeast system can be alternatively used to initially screen for any drugs that can help rescue the protein levels of asparagine synthetase before applying them to further studies in mammals and humans. Furthermore, this mutation might specifically be introduced into the asparagine synthetase gene of the target cancer cells in order to suppress the overproduction of asparagine synthetase within these abnormal cells, therefore inhibiting the growth of cancer, which might be helpful for patients with blood cancer to prevent them developing any resistance to the conventional asparaginase treatment.

**Abstract:**

Asparagine synthetase deficiency (ASD) has been found to be caused by certain mutations in the gene encoding human asparagine synthetase (ASNS). Among reported mutations, A6E mutation showed the greatest reduction in ASNS abundance. However, the effect of A6E mutation has not yet been tested with yeast asparagine synthetase (Asn1/2p). Here, we constructed a yeast strain by deleting *ASN2* from its genome, introducing the A6E mutation codon to *ASN1*, along with *GFP* downstream of *ASN1*. Our mutant yeast construct showed a noticeable decrease of Asn1p(A6E)-GFP levels as compared to the control yeast expressing Asn1p(WT)-GFP. At the stationary phase, the A6E mutation also markedly lowered the assembly frequency of the enzyme. In contrast to Asn1p(WT)-GFP, Asn1p(A6E)-GFP was insensitive to changes in the intracellular energy levels upon treatment with sodium azide during the log phase or fresh glucose at the stationary phase. Our study has confirmed that the effect of A6E mutation on protein expression levels of asparagine synthetase is common in both unicellular and multicellular eukaryotes, suggesting that yeast could be a model of ASD. Furthermore, A6E mutation could be introduced to the *ASNS* gene of acute lymphoblastic leukemia patients to inhibit the upregulation of ASNS by cancer cells, reducing the risk of developing resistance to the asparaginase treatment.

## 1. Introduction

Being one of the fundamental processes required for life and survival, the metabolism of any living organism is kept running through the action and network of metabolic enzymes in a well-coordinated and tightly-regulated manner. The enzyme activity and pathway flux can be regulated through several well-known molecular and cellular processes, such as transcriptional and post-transcriptional controls [1,2,3], protein homeostatic turnover (balance between synthesis and degradation) [4,5], phosphorylation and dephosphorylation [2,6], and sequestration or translocation to different subcellular compartments [7,8,9]. Interestingly, attention is currently being focused on metabolic regulation via the supramolecular assembly or high-order structure formation of metabolic enzymes into micron-sized structures within living cells, as has been revealed in several species [10,11,12,13,14,15,16,17,18]. The highly dynamic and reversible assembly and disassembly of metabolic enzymes has been suggested as an intracellular mechanism to either stimulate or suppress the activity of the target metabolic enzymes, allowing the modulation of their cognate metabolic flux to reach the desired metabolic state and cellular outcome [10,11,14,19,20,21,22,23,24,25].

Asparagine synthetase is also one of metabolic enzymes capable of microscopically visible structure formation [11,17,18,26,27,28]. Its biochemical function is to convert L-aspartate to L-asparagine in an ATP-dependent fashion. Regarding its molecular structure, the N-terminal region is home to the glutamine amidotransferase (GAT) domain, which is responsible for L-glutamine hydrolysis to obtain ammonia (NH_3_) molecules. The synthetase domain is found at the C-terminus of the enzyme, where L-aspartate and Mg-ATP are catalyzed to form the intermediate, β-aspartyl-AMP. After the nucleophilic attack of β-aspartyl-AMP by NH_3_, transferred from the GAT domain through the molecular tunnel connecting between the two domains, L-asparagine can then be synthesized [29]. Recently, our mutation study using yeast as a model organism suggested that the assembly–disassembly feature of asparagine synthetase is coupled to the regulation and status of its enzymatic activity [28]. Interestingly, human asparagine synthetase has been found to cluster around the centrosome and the mitotic spindle in the dividing cells [27]. The artificial yeast constructs expressing nuclear-targeted and loss-of-function asparagine synthetase showed that the association between the enzyme and the mitotic spindle is not activity-dependent, implying that asparagine synthetase has an unprecedented function in the cell division process, in addition to its well-known function in cellular metabolism [30].

In humans, several mutations in the gene coding for asparagine synthetase (ASNS) have been identified as being associated with asparagine synthetase deficiency (ASD), resulting in neurological problems and microcephaly [31,32,33,34,35,36,37,38,39,40,41,42,43,44,45,46,47]. Among these reported mutations, the change of alanine to glutamate at the 6th amino acid residue of the enzyme (A6E) has been demonstrated to have the most negative impact on the protein expression levels of human asparagine synthetase [31]. However, the A6E mutation, originally identified in the human study, has not yet been tested in the yeast system. As shown by the very first case report of ASD [31], the A6E mutation reduced the protein expression of human ASNS to much lower than usual. Here, we suspect the A6E mutation could affect the protein expression levels and also alter the assembly capability of yeast asparagine synthetase.

To address this, we designed and engineered the yeast genome by first knocking out *ASN2* (one of the two duplicated genes responsible for yeast asparagine biosynthesis; this was to simplify our study, as the presence of wild-type Asn2p can interfere with the mutation effect on assembly feature of Asn1p) and then introducing the A6E mutation codon (GCC → GAA) to the chromosomal *ASN1*, together with *GFP* (downstream of *ASN1*, for both determination of protein expression and direct visualization under fluorescence microscope). Therefore, (1) the protein expression levels and (2) the supramolecular assembly frequency of Asn1p(A6E)-GFP (expressed by yeast *asn1*(*A6E*)*::GFP* (*asn2*Δ)) could then be compared with those of the control, Asn1p(WT)-GFP (expressed by yeast *ASN1*(*WT*)*::GFP* (*asn2*Δ)). Our findings suggested yeast as a model in the search for certain novel treatments for ASD. In addition, the A6E mutation codon could be incorporated into the *ASNS* gene of patients with acute lymphoblastic leukemia to minimize the risk of developing a resistance to the asparaginase treatment, as A6E mutation can block the attempt at ASNS overexpression by the cancer cells.

## 2. Materials and Methods

### 2.1. Bacteria, Yeast, Growth and Selection Media, and Oligonucleotides

*Escherichia coli* One Shot™ MAX Efficiency™ DH5α-T1^R^ (Thermo Fisher Scientific, Waltham, MA, USA) was used as bacterial host for transformation and propagation of the recombinant plasmids. LB medium (0.5% (*w*/*v*) yeast extract (BD Biosciences, San Jose, CA, USA), 1% (*w*/*v*) Bacto^TM^ Tryptone (BD Biosciences), and 1% (*w*/*v*) sodium chloride (BDH Prolabo, Leicestershire, England)) supplemented with 100 μg/mL ampicillin (PanReac AppliChem, Darmstadt, Germany) was used for selection. Bacterial cultures were maintained at 37 °C.

Yeast *BY4741* (*MATa his3*Δ*1 leu2*Δ*0 met15*Δ*0 ura3*Δ*0*) (Thermo Fisher Scientific) was used as a background strain for yeast chromosomal gene modifications. YPD medium (1% (*w*/*v*) yeast extract, 2% (*w*/*v*) Bacto^TM^ Peptone (BD Biosciences), and 2% (*w*/*v*) dextrose (Sigma-Aldrich, St. Louis, MO, USA)) was used for general yeast cultures. G418 (PanReac AppliChem) (400 μg/mL final concentration) and hygromycin B (Merck, Darmstadt, Germany) (200 μg/mL final concentration) were used to select yeast transformants as indicated. All yeast strains were maintained at 30 °C.

All primers used in PCR-based, site-directed mutagenesis, amplification of DNA cassettes for transformation of yeast, and yeast strain verification by PCR and DNA sequencing are shown in Table S1.

### 2.2. Construction of Yeast asn2Δ

PCR-based engineering of yeast genome [48] was used as the molecular approach to delete *ASN2* from the genome of yeast *BY4741*. Additionally, pFA6a-hphMX6 (Addgene, a gift from J. Wilhelm, UC San Diego, CA, USA) was used as the plasmid template to create the *asn2*Δ*::hygR* DNA cassette carrying (from 5′ to 3′) 50 bp upstream of the *ASN2* start codon, hygromycin resistance gene, and 50 bp downstream of the *ASN2* stop codon. The PCR reaction was set up using a KOD Hot Start DNA Polymerase Kit (Merck). Yeast *BY4741* was transformed with the purified *asn2*Δ*::hygR* DNA cassette using lithium acetate (Sigma-Aldrich)–single stranded DNA (Sigma-Aldrich)–polyethylene glycol (Sigma-Aldrich) and heat shock method. YPD supplemented with hygromycin B (200 μg/mL final concentration) was used for selection. The genomic DNA of each positive clone was extracted and verified via PCR to examine whether *ASN2* had successfully been deleted from the yeast genome.

### 2.3. Construction of Plasmid pFA6a-asn1(A6E)-GFP-kanMX6

The plasmid pFA6a-ASN1-GFP-kanMX6, which had been previously constructed [28], was used as the plasmid template to introduce the A6E mutation codon (GCC → GAA) to the *ASN1* coding sequence within this plasmid via PCR-based, site-directed mutagenesis. The mutagenic primers were 5′-phosphorylated using a T4 Polynucleotide Kinase Kit (New England Biolabs, Ipswich, MA, USA), following the manufacturer’s instructions. The PCR reaction was set up using a KOD Hot Start DNA Polymerase Kit. The parental plasmid was removed from the mutagenized PCR products by *Dpn*I (Thermo Fisher Scientific) treatment. The mutagenized PCR products (linear form) were then purified using a GenepHlow^TM^ Gel/PCR Kit (Geneaid, New Taipei City, Taiwan). The ligation was performed to circularize purified PCR products using a T4 DNA Ligase Kit (Thermo Fisher Scientific). *Escherichia coli* One Shot™ MAX Efficiency™ DH5α-T1^R^ competent cells were transformed with the ligation products using the heat shock method. The bacterial transformants were grown on LB agar supplemented with ampicillin (100 μg/mL final concentration). Plasmid DNA was isolated from each randomly picked transformant using a Presto™ Mini Plasmid Kit (Geneaid) and subsequently verified by plasmid sequencing (Macrogen, Seoul, Korea). The resulting plasmid after mutagenization was referred to as pFA6a-asn1(A6E)-GFP-kanMX6.

### 2.4. Construction of Yeast asn1(A6E)::GFP (asn2Δ)

Yeast *asn1(A6E)::GFP* (*asn2*Δ) was created for characterization of the protein expression levels and the assembly frequency of Asn1p(A6E)-GFP, in comparison with those of Asn1p(WT)-GFP, which was expressed by the control yeast *ASN1(WT)::GFP* (*asn2*Δ), as previously constructed [28]. For this, yeast *asn2*Δ, constructed from the very first step, was used as the base strain. The sequence-verified plasmid pFA6a-asn1(A6E)-GFP-kanMX6 was used as DNA template to make the DNA cassette harboring (from 5′ to 3′) 50 bp upstream of *ASN1* start codon, *asn1*(*A6E*) coding sequence, *GFP*, kanamycin resistance gene, and 50 bp downstream of *ASN1* stop codon. The PCR reaction was performed using the KOD Hot Start DNA Polymerase Kit. Yeast *asn2*Δ was transformed with the purified DNA cassette of *asn1*(*A6E*)*::GFP;kanR* using lithium acetate–single-stranded DNA–polyethylene glycol and the heat shock method. Yeast transformants were selected on the YPD agar supplemented with G418 (400 μg/mL final concentration) and hygromycin B (200 μg/mL final concentration). The yeast transformants were initially screened under the fluorescence microscope (to check for GFP signals) and their genomic DNA samples were then extracted to prepare PCR products for DNA sequencing (Macrogen).

### 2.5. Western Blot Analysis

Yeast *asn1*(*A6E*)*::GFP* (*asn2*Δ) was grown in liquid YPD at 30 °C with shaking to the indicated growth stages to monitor the protein expression levels of Asn1p(A6E)-GFP in comparison with those of Asn1p(WT)-GFP, as expressed by the control yeast strain *ASN1*(*WT*)*::GFP* (*asn2*Δ). At each stage of growth, the optical density at 600 nm (OD_600_/_mL_) was measured for each strain or clone in order to ensure all yeast samples had the same amount of cells during protein sample preparation (taking 1 OD_600_ cells to prepare whole-cell extracts from log-phase cultures, 5 OD_600_ cells from 1-day cultures, and 10 OD_600_ cells from 5-day cultures).

After centrifugation at 6000 rpm for 3 min and removal of supernatant, cells were lysed in 100 μL SDS-PAGE sample buffer containing 4M urea (Merck), (1:20) beta-mercaptoethanol (PanReac Applichem), and (1:1000) protease inhibitor cocktail (Sigma-Aldrich). About 50 μL glass beads (425–600 μm) (Sigma-Aldrich) was added to help in cell lysis during the vigorous vortexing for 1 min. After boiling protein samples at 95 °C for 10 min and placing them on ice for 5 min, they were centrifuged at 10,000 rpm for 1 min at room temperature and stored at −25 °C until use.

A 10 μL aliquot of each protein sample was resolved using 8% SDS-PAGE (Bio-Rad, Hercules, CA, USA). BLUeye Prestained Protein Ladder (Sigma-Aldrich) was used to identify the approximate sizes of Asn1p(A6E)-GFP, Asn1p(WT)-GFP, and loading control phosphoglycerate kinase (Pgk1p). The resolved proteins were transferred to the PVDF membrane (Bio-Rad) using a Trans-Blot^®^ SD Semi-Dry Transfer Cell (Bio-Rad). Each membrane was cut into 2 pieces between 75 and 63 kDa bands of the pre-stained protein ladder. The upper piece of each divided blot was used to detect GFP-tagged Asn1p with anti-GFP (91.7 kDa for Asn1p(WT)-GFP, 91.8 kDa for Asn1p(A6E)-GFP). The lower piece of each divided blot was used to detect Pgk1p (as internal loading control) (44.7 kDa). Western blotting was performed using the standard protocol. For detection of GFP-tagged Asn1p, (1:5000) rabbit polyclonal anti-GFP (A01388, GenScript, Piscataway, NJ, USA) was used as the primary antibody and (1:5000) HRP-conjugated goat anti-rabbit IgG was used as the secondary antibody (31460, Thermo Fisher Scientific). For detection of internal loading control, (1:10,000) mouse monoclonal anti-phosphoglycerate kinase (Pgk1p) (clone 22C5D8, 459250, Thermo Fisher Scientific) was used as the primary antibody and (1:5000) HRP-conjugated goat anti-mouse IgG (62-6520, Thermo Fisher Scientific) was used as the secondary antibody. A Western Blotting Detection System (GE Healthcare, Piscataway, NJ, USA) was used to develop the chemiluminescent signals before exposure to X-ray films (Fujifilm, Tokyo, Japan). Three independent experiments were performed to confirm the results. The quantification was performed using ImageJ (NIH). The expression levels of asparagine synthetase were normalized by those of the corresponding phosphoglycerate kinase before the fold change of protein expression between the A6E mutant and WT enzyme was made.

### 2.6. Growth Curve Analysis

Two yeast strains, *asn1*(*A6E*)::*GFP* (*asn2*Δ) and *ASN1*(*WT*)*::GFP* (*asn2*Δ), were diluted in fresh YPD to give the starting OD_600_ ~ 0.1 and continuously cultured at 30 °C with shaking for 5 days. An aliquot was taken out from each culture to measure OD_600_ using an Evolution™ 300 UV-Vis Spectrophotometer (Thermo Fisher Scientific, Waltham, MA, USA) at the following time points; 4.5, 24, 48, 72, 96, and 120 h, respectively. The data were used to plot their growth curve and statistically analyzed (paired *t*-test) using GraphPad Prism Version 9.0.2 (161) (GraphPad, San Diego, CA, USA).

### 2.7. In Vivo Assembly Frequency Assay of Asn1p(A6E)-GFP vs. Asn1p(WT)-GFP

The assembly frequencies of both Asn1p(A6E)-GFP and Asn1p(WT)-GFP were characterized by growing yeast *asn1*(*A6E*)::*GFP* (*asn2*Δ) and control yeast *ASN1*(*WT*)*::GFP* (*asn2*Δ) in liquid YPD at 30 °C with shaking, to 3 different growth stages: log phase (4.5 h), saturation (1 day), and stationary phase (5 days). At each stage of growth, the cells of both strains (two different clones for each) were fixed with formaldehyde (1:10 (*v*/*v*) 37% formaldehyde to cell culture) for 15 min at room temperature with shaking, washed twice with sterile water, and resuspended in 1M sorbitol prior to counting the cells under the fluorescence microscope (Zeiss Apotome.2 optical sectioning structured illumination system using Plan-NEOFLUAR 100x/1.3 Oil objective (Zeiss, Jena, Germany), the Advanced Cell Imaging Center, Institute of Molecular Biosciences) to obtain the frequency of cells with Asn1p-GFP structures (filaments or foci). For each experiment, 5 different fields of view (about 50 cells/field of view) were inspected and the data were reported as % cells with Asn1p-GFP structures ((number of cells showing Asn1p-GFP filaments or foci)/(total number of cells counted) *100). Three independent experiments were performed for mutant (*asn1*(*A6E*)::*GFP* (*asn2*Δ)) and WT (*ASN1*(*WT*)*::GFP* (*asn2*Δ)) strains to finally obtain the average percentage ± SEM. Statistical analysis (paired *t*-test) was performed using GraphPad Prism Version 9.0.2 (161).

### 2.8. Sodium Azide Treatment of Log-Phase Yeast Cultures

To test whether depletion of intracellular ATP would show any effect on the assembly frequency of Asn1p(A6E)-GFP and Asn1p(WT)-GFP, the diluted cultures (OD_600_ ~ 0.1–0.2) of two yeast strains, *asn1*(*A6E*)::*GFP* (*asn2*Δ) and *ASN1*(*WT*)*::GFP* (*asn2*Δ), were grown in liquid YPD at 30 °C with shaking to log phase (OD_600_ ~ 0.4–0.6). A 990-μL aliquot of each log-phase culture was transferred to a microcentrifuge tube and 10 μL of 1 M sodium azide (10 mM final concentration) was added to the tube prior to incubation for 15 min at room temperature with shaking. For the untreated controls, 10 μL of sterile water was added to each log-phase culture instead. The cells were then fixed with formaldehyde using the same protocol as mentioned above. After washing the fixed cells with sterile water twice and resuspending them in 1M sorbitol, wet slides were prepared to observe the assembly frequency of Asn1p(A6E)-GFP and Asn1p(WT)-GFP under NaN_3_-treated and untreated conditions, using the same protocol as mentioned above. Three independent experiments were performed for each strain or condition and the data were reported as average percentages ± SEM. Statistical analysis (paired *t*-test) was performed using GraphPad Prism Version 9.0.2 (161).

### 2.9. Glucose Addition to 5-Day Yeast Cultures

To test whether the addition of fresh glucose can trigger the disassembly of Asn1p(A6E)-GFP and Asn1p(WT)-GFP structures formed in the yeast cells at the stationary phase, two yeast strains, *asn1*(*A6E*)::*GFP* (*asn2*Δ) and *ASN1*(*WT*)*::GFP* (*asn2*Δ), were grown in liquid YPD at 30 °C with shaking for 5 days. A 950-μL aliquot of each 5-day culture was transferred to a microcentrifuge tube and 50 μL of 40% (*w*/*v*) glucose solution (2% (*w*/*v*) final concentration) was added to the tube prior to incubation for 15 min at room temperature with shaking. For the untreated controls, 50 μL of sterile water was added to each 5-day culture, instead. Glucose-treated and untreated cells of each strain were fixed with formaldehyde, as mentioned above. Wet slides of each sample were prepared using the same protocol as mentioned above. Three independent experiments were performed for each strain or condition and the data were reported as average percentages ± SEM. Statistical analysis (paired *t*-test) was performed using GraphPad Prism Version 9.0.2 (161).

### 2.10. Cell Imaging

Fixed cells were prepared using the same protocol as mentioned above. Fixed cells resuspended in 1M sorbitol (Sigma-Aldrich) were then dropped onto microscopic slides (Shandon SuperFrost Plus, Thermo Fisher Scientific). After putting on the coverslips (Menzel Gläser, Thermo Fisher Scientific), excess liquid was blotted off with a lint-free lab wipe to prevent cells from floating around, then the edges of the coverslips were sealed with nail polish. Cell imaging (Zeiss LSM800 with AiryScan using Plan-APOCHROMAT 63X/1.4 Oil DIC ∞/0.17 objective (Zeiss, Jena, Germany), the Advanced Cell Imaging Center, Institute of Molecular Biosciences) was performed by capturing the yeast samples in Z-stack of approximately 1–3 μm and compressing each set of Z-stack images into a single 2-dimensional image using maximum projection (ZEN 2.1 blue edition, Zeiss, Jena, Germany).

## 3. Results and Discussion

### 3.1. A6E Mutation Causes a Lowering in the Protein Expression Levels of Asparagine Synthetase

**Figure 1:**
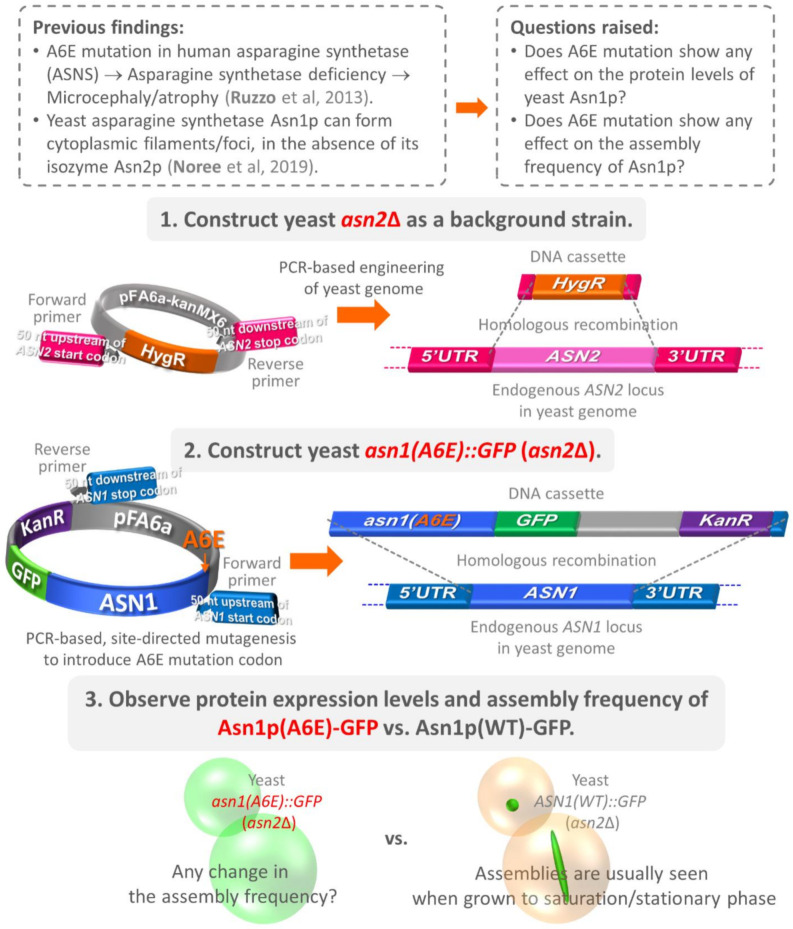
Illustration of the background information, questions, and experimental design used in this study. Previous studies have found that asparagine synthetase deficiency is caused by certain mutations in the human *ASNS* gene, including A6E, resulting in a microcephaly phenotype. In yeast, asparagine synthetases Asn1p and Asn2p are able to form cytoplasmic foci or filaments. However, only Asn1p can self-assemble independently in the absence of Asn2p, but not vice versa. Therefore, in this study, we investigated whether the introduction of the A6E mutation to yeast Asn1p can lower the protein levels of the yeast enzyme, as has been observed in humans. To do this, we decided to knock out *ASN2* from the yeast genome (to prevent complications in data analysis, as Asn1p and Asn2p can form heteromeric proteins), then introduced the A6E mutation codon to the chromosomal *ASN1*, along with *GFP* (downstream of *ASN1*). The yeast construct expressing Asn1p(A6E)-GFP can eventually be characterized by the protein levels and assembly frequency of the mutant enzyme as compared with the control yeast expressing Asn1p(WT)-GFP. Abbreviations: A6E = mutation change from alanine to glutamate at the 6th amino acid residue of the enzyme; *ASN1* = yeast asparagine synthetase 1 (gene); Asn1p = yeast asparagine synthetase 1 (enzyme); *ASN2* = yeast asparagine synthetase 2 (gene); Asn2p = yeast asparagine synthetase 2 (enzyme); GFP = green fluorescent protein; *HygR* = hygromycin resistance gene; *KanR* = kanamycin resistance gene; UTR = untranslated region; WT = wild-type.

A6E mutation was originally reported as one of the mutations associated with ASD, as this mutation adversely affects the protein expression levels of asparagine synthetase, consequently causing neurological defects and small head and brain size in humans [31]. We suspected this mutation would also show a similar effect in other eukaryotic species. Therefore, in our study, we decided to test the effects of A6E mutation with one of the genes encoding asparagine synthetase in budding yeast. Differing from their human homolog (*ASNS* is the only gene responsible for asparagine biosynthesis in human cells), yeast asparagine synthetases Asn1p and Asn2p can be encoded by two evolutionarily duplicated genes, *ASN1* and *ASN2*, respectively. Furthermore, Asn1p and Asn2p are both capable of supramolecular assembly in vivo (for the amino acid sequence alignment of human and yeast asparagine synthetases, see Figure S1). In our previous study and others [17,28], it was found that the assembly of Asn1p into visible intracellular structures is independent of the availability of Asn2p, but not vice versa. Therefore, we decided to delete *ASN2* from the yeast genome, allowing only *ASN1* to be mutated and characterized (Figure 1).

**Figure 2:**
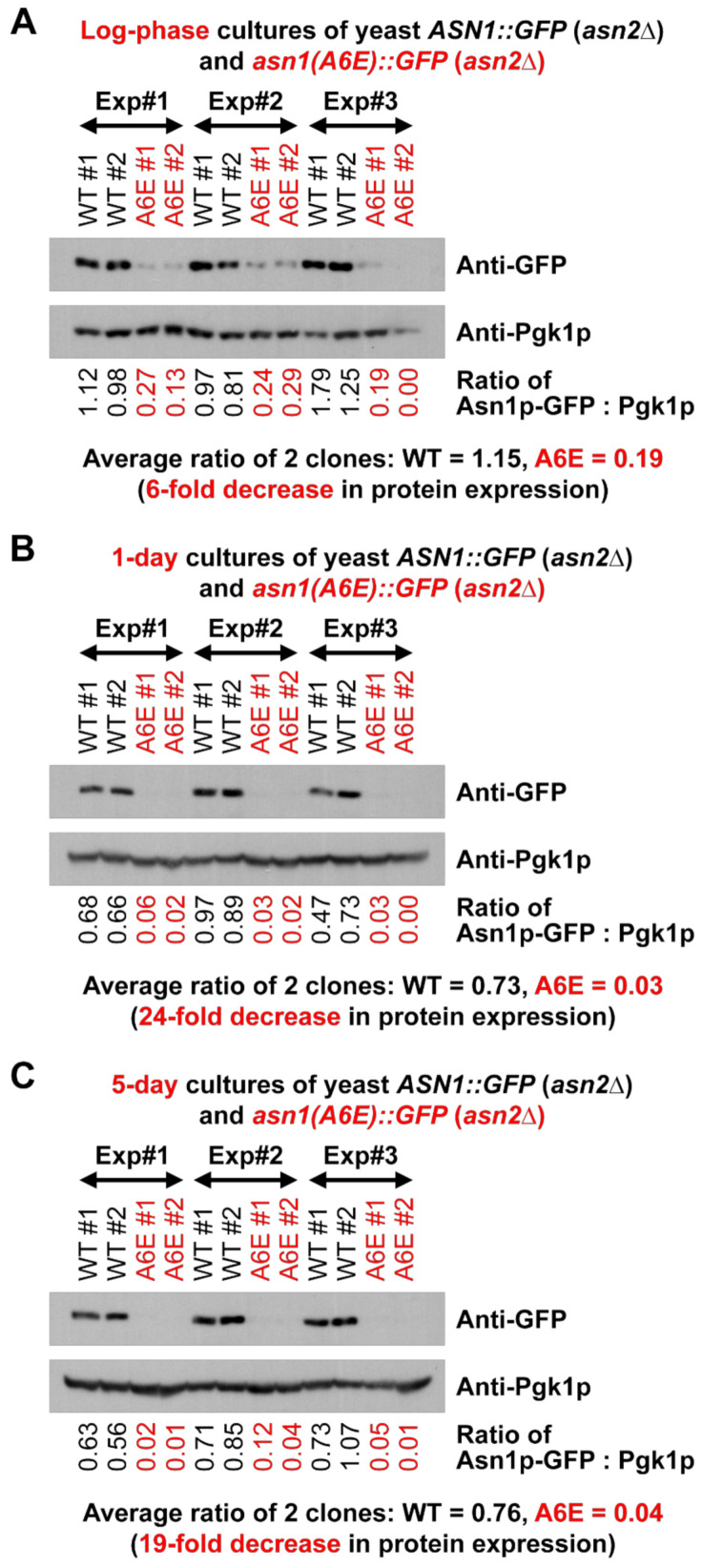
The A6E mutation adversely affects the protein expression levels of yeast asparagine synthetase Asn1p. Two different clones of yeast *ASN1*(*WT*)*::GFP* (*asn2*Δ) and *asn1*(*A6E*)*::GFP* (*asn2*Δ) were grown in liquid YPD at 30 °C with shaking to log phase (**A**), saturation (1-day cultures) (**B**), and stationary phase (5-day cultures) (**C**). Then, 1, 5, and 10 OD_600_ cells for log-phase, 1-day, and 5-day cultures, respectively, were taken to prepare whole cell extracts for SDS-PAGE and Western blot analysis. Each membrane was cut into 2 pieces between 75 and 63 kDa bands of the pre-stained protein ladder. The upper piece of each divided blot was used to detect GFP-tagged Asn1p with anti-GFP (91.7 kDa for Asn1p(WT)-GFP and 91.8 kDa for Asn1p(A6E)-GFP). The lower piece of each divided blot was used to detect Pgk1p (as internal loading control) (44.7 kDa). Three independent experiments were set up to confirm the results. The quantification was then performed using ImageJ. The protein expression levels of asparagine synthetase were normalized by those of the corresponding phosphoglycerate kinase before the fold change of protein expression between the A6E mutant and WT enzyme was made. Full blots are shown in Figure S2. Abbreviations: A6E = yeast expressing Asn1p(A6E)-GFP (*asn2*Δ); Exp = experiment; Pgk1p = yeast phosphoglycerate kinase (enzyme); WT = yeast expressing Asn1p(WT)-GFP (*asn2*Δ).

After engineering the genome, the yeast strain *asn1*(*A6E*)*::GFP* (*asn2*Δ) was successfully constructed. First, as we hypothesized that the A6E mutation might affect the protein expression levels of yeast asparagine synthetase, similar to the previous report in humans [31], we performed the Western blot analysis of two yeast strains, *asn1*(*A6E*)*::GFP* (*asn2*Δ) and *ASN1*(*WT*)*::GFP* (*asn2*Δ), cultured to 3 different growth stages, including the log phase, saturation (1 day), and stationary phase (5 days). From our quantification, the normalized protein expression levels of asparagine synthetase with the A6E mutation, Asn1p(A6E)-GFP, showed approximately 6-fold decrease (log-phase), 24-fold decrease (saturation), and 19-fold decrease (stationary phase), as compared with those of the wild-type enzyme, Asn1p(WT)-GFP (Figure 2A–C). Therefore, we concluded that the protein expression levels of Asn1p-GFP are negatively affected by A6E mutation.

### 3.2. Asparagine Synthetase with A6E Mutation Also Affects the Growth of Yeast

**Figure 3:**
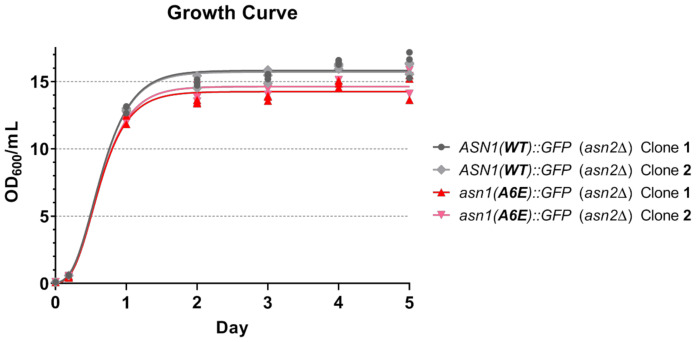
Yeast grows slower than usual when asparagine synthetase carries the A6E mutation. Two different clones of yeast *ASN1*(*WT*)*::GFP* (*asn2*Δ) and *asn1*(*A6E*)*::GFP* (*asn2*Δ) were grown in liquid YPD at 30 °C with shaking (starting OD_600_ ~ 0.1). An aliquot of each culture was taken out to measure OD_600_ during the log phase, day 1, day 2, day 3, day 4, and day 5, respectively. Three independent experiments were performed to confirm the results and for statistical analysis. Their growth curve was plotted and statistical analyses were tested (paired *t*-test, 99% confidence level) using GraphPad Prism. Numerical data, used for creating the growth curve, and a summary of statistical analyses are shown in Table 1. Abbreviation: OD_600_/_mL_ = optical density (per milliliter) at 600 nm.

Since microcephaly (small head and brain size) in humans has been reported to be associated with asparagine synthetase deficiency (ASD), we suspected that the A6E mutation affecting protein expression levels of yeast asparagine synthetase would also have negative effects on the overall growth of yeast. To address this question, we observed the optical density at 600 nm (OD_600_) of two yeast strains, *asn1*(*A6E*)*::GFP* (*asn2*Δ) and *ASN1*(*WT*)*::GFP* (*asn2*Δ), cultured in rich medium (YPD) at 30 °C with shaking, by following their growth starting from the diluted cultures with the same initial OD_600_ (~0.1). We found that yeast expressing Asn1p with A6E mutation did show significantly slower growth than yeast expressing the wild-type Asn1p (Figure 3, Table 1). From our previous study with other mutations (including C1A and R344A, which can disrupt the biochemical activity of the GAT domain and synthetase domain, respectively, of yeast asparagine synthetase Asn1p), none of the C1A, R344A, or C1A-R344A mutants showed any growth difference from the control yeast *ASN1*(*WT*)*::GFP* (*asn2*Δ) [28]. Therefore, our current study revealed A6E as a critical mutation that affects both the protein expression levels of asparagine synthetase and the overall growth of yeast.

### 3.3. The Assembly Frequency of Asn1p(A6E)-GFP Is Significantly Lower Than That of Asn1p(WT)-GFP and the Mutant Enzyme Does Not Show Rapid Response to Changes in the Intracullar Energy Levels

**Figure 4:**
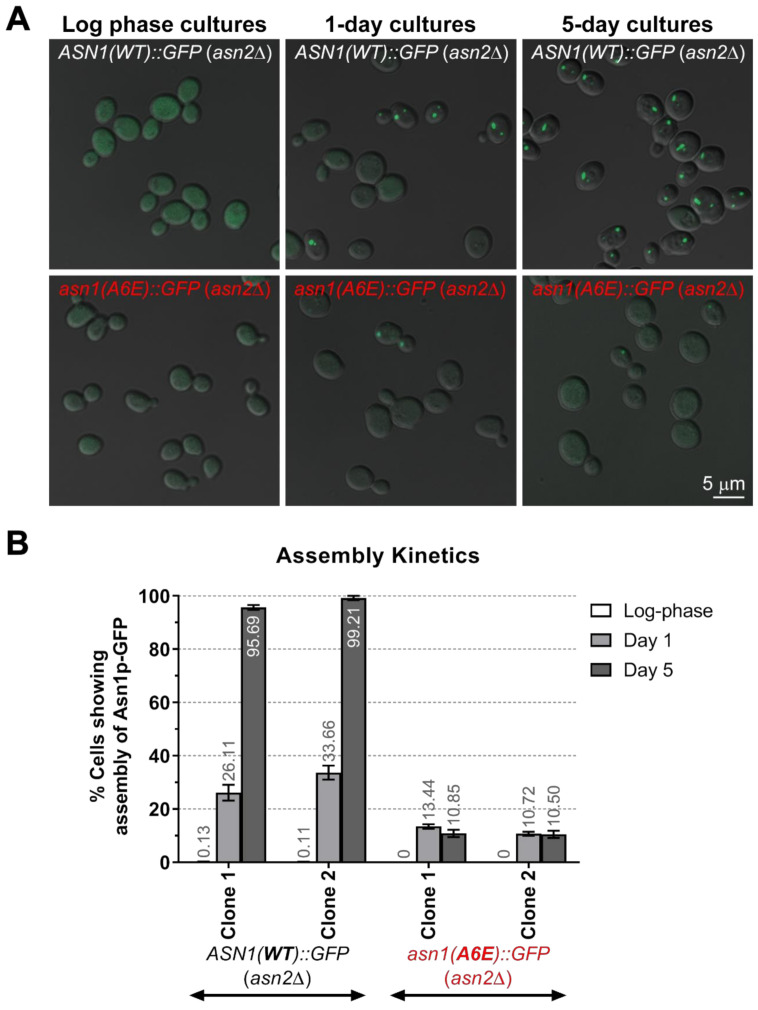
Assembly frequency of asparagine synthetase Asn1p is affected by A6E mutation. Two different clones of yeast *ASN1*(*WT*)*::GFP* (*asn2*Δ) and *asn1*(*A6E*)*::GFP* (*asn2*Δ) were grown in liquid YPD at 30 °C with shaking to the indicated cultured conditions for imaging (**A**) and to study assembly kinetics (**B**). Representative confocal images of yeast grown to 3 different stages of growth are shown in (**A**). Assembly kinetics of Asn1p(WT)-GFP vs. Asn1p(A6E) were investigated at log phase, day 1, and day 5, respectively. At day 5, the assembly frequency of Asn1p(A6E)-GFP (10.5–10.9%) could not be increased to similar levels to that of Asn1p(WT)-GFP (95.7–99.2%) (**B**). Three independent experiments were performed and the data are reported as averages ± SEM. The graph was plotted and statistical analyses were performed using GraphPad Prism. Numerical data used to plot this graph and a summary of statistical analyses are shown in Table S2.

We had determined that the A6E mutation does affect the protein expression levels of yeast asparagine synthetase, similar to the previous results shown in humans [31]. Next, we characterized whether the yeast Asn1p carrying A6E mutation would show any change in its assembly frequency profile when compared to the wild-type Asn1p. Interestingly, the assembly frequency of Asn1p(A6E)-GFP was significantly lower than that of Asn1p(WT)-GFP when growing yeast cells to stationary phase (A6E: 10.9% and 10.5% vs. WT: 95.7% and 99.2%, average of 3 independent experiments for two different clones) (Figure 4A,B and Table S2).

**Figure 5:**
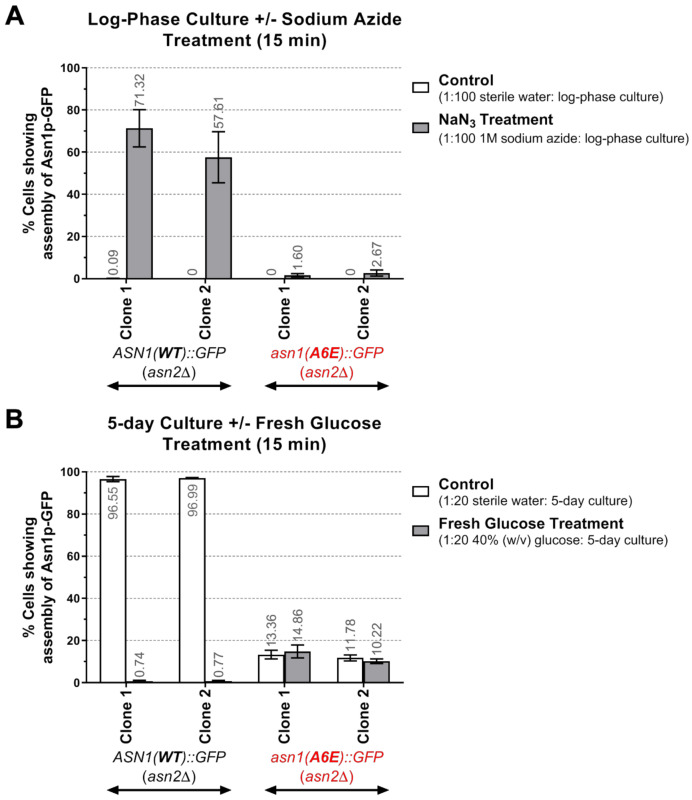
Assembly of Asn1p(A6E)-GFP is unresponsive to changes in the intracellular ATP levels. Two different clones of yeast *ASN1*(*WT*)*::GFP* (*asn2*Δ) and *asn1*(*A6E*)*::GFP* (*asn2*Δ) were grown in liquid YPD at 30 °C with shaking to the indicated cultured conditions to test sodium azide treatment (**A**) and fresh glucose treatment (**B**). Sodium azide (10 mM final concentration) was used to treat log-phase yeast cultures for 15 min at room temperature with shaking. The assembly frequency of Asn1p(WT)-GFP can be triggered from a range of 0–0.1% (control treatment) to a range of 57.6–71.3% (after 15 min treatment with sodium azide), while the assembly frequency of Asn1p(A6E)-GFP was not significantly altered (control treatment: 0% vs. sodium azide treatment: 1.6–2.7%) (**A**). Fresh glucose (2% (*w*/*v*) final concentration) was added to the 5-day yeast cultures for 15 min at room temperature with shaking. Asn1p(WT)-GFP structures rapidly disappeared (control treatment: 96.6–97.0% vs. glucose treatment: 0.7–0.8%), whereas Asn1p(A6E)-GFP structures were not sensitive to the addition of fresh glucose (control treatment: 11.8–13.4% vs. glucose treatment: 10.2–14.9%) (**B**). Three independent experiments were performed and the data are reported as averages ± SEM. Graphs were plotted and statistical analyses were performed using GraphPad Prism. Numerical data used to plot these graphs and a summary of statistical analyses are shown in Table S2.

In addition, once we treated the log phase yeast cultures with sodium azide (used to deplete the intracellular ATP) for 15 min, we found that while yeast *ASN1*(*WT*)*::GFP* (*asn2*Δ) exhibited a rapid increase of enzyme assembly (57.6–71.3% cells with Asn1p(WT)-GFP structures), yeast *asn1*(*A6E*)*::GFP* (*asn2*Δ), in contrast, showed only a few cells (1.6–2.7%) with Asn1p(A6E)-GFP assemblies (Figure 5A, Table S2).

We also tested whether a 15 min treatment of the 5-day yeast cultures with fresh glucose (2%(*w*/*v*) final concentration) would have any effect on the assembly of the enzyme. As expected, yeast *ASN1*(*WT*)*::GFP* (*asn2*Δ) showed rapid disassembly of Asn1p(WT)-GFP, having only 0.7–0.8% cells with Asn1p(WT)-GFP structures (compared with its control group, which still had 96.6–97.0% cells with Asn1p(WT)-GFP structures), whereas yeast *asn1*(*A6E*)*::GFP* (*asn2*Δ) did not show any active response to the addition of fresh glucose (treated group: 10.2–14.9% vs. control group: 11.8–13.4% cells with Asn1p(A6E)-GFP assemblies) (Figure 5B, Table S2).

## 4. Conclusions

Altogether, in this study we have shown that the A6E mutation not only adversely affects the abundance of yeast asparagine synthetase, leading to slower growth than usual, but also the assembly feature of the enzyme, making their supramolecular structures insensitive to changes in the intracellular energy levels. As A6E mutation does show a common effect on the protein expression of asparagine synthetase in both yeast and humans, this suggests that yeast could be an alternative model of ASD, as already seen in several studies using yeast as models of amyloid and neurodegenerative diseases [49,50,51,52,53,54,55,56,57,58].

Additionally, patients with acute lymphoblastic leukemia (ALL) typically receive asparaginase treatment to lower asparagine levels in order to block the growth and survival of cancer cells. However, in some cases, the patients later develop a resistance to the treatment as the cancer cells fight back by upregulating the ASNS expression to rescue the asparagine levels [59,60]. The introduction of the A6E mutation to the *ASNS* gene could be an alternative approach to impede the attempt of cancer cells to stimulate the expression of ASNS, such that the ALL patients would not develop any resistance to the asparaginase treatment. However, this idea and experimental design needs to be tested carefully, making sure that it will not show any adverse side effects.

## Figures and Tables

**Table 1:** Data for OD_600_ measurements and statistical analysis of two yeast strains, *ASN1(WT)::GFP* (*asn2*Δ) and *asn1(A6E)::GFP* (*asn2*Δ), used to plot the growth curve shown in Figure 3. Two different clones of each strain were cultured in YPD at 30 °C with shaking (starting OD_600_ ~ 0.1). Their OD_600_/_mL_ values were measured at 4.5 h (log phase), day 1, day 2, day 3, day 4, and day 5, respectively. Three independent experiments were performed and are reported as averages ± SEM. Abbreviations: SEM = standard error of the mean; YPD = yeast culture (rich) medium.

Yeast Strain	Clone #	OD_600_/mL (Average ± SEM)					
		Log phase	Day 1	Day 2	Day 3	Day 4	Day 5
*ASN1(**WT**)::GFP* (*asn2*Δ)	1	0.60 ± 0.01	12.97 ± 0.16	14.91 ± 0.13	15.39 ± 0.11	16.42 ± 0.10	16.35 ± 0.57
	2	0.60 ± 0.01	12.89 ± 0.06	15.12 ± 0.31	15.40 ± 0.29	16.12 ± 0.11	16.07 ± 0.26
*asn1(**A6E**)::GFP* (*asn2*Δ)	1	0.50 ± 0.04	12.03 ± 0.22	13.48 ± 0.08	13.79 ± 0.11	14.82 ± 0.15	14.78 ± 0.57
	2	0.51 ± 0.03	12.18 ± 0.14	14.01 ± 0.36	14.30 ± 0.13	14.95 ± 0.18	15.12 ± 0.53
**Paired *t*-Test (99% confidence level, two-tailed)**					***p*** **-value**	***p*** **-value Summary**	**Significant different?** **(*p* < 0.01)**
*ASN1(**WT**)::GFP* (*asn2*Δ) Clone **1** vs. *ASN1(**WT**)::GFP* (*asn2*Δ) Clone **2**					0.3897	ns	No
*ASN1(**WT**)::GFP* (*asn2*Δ) Clone **1** vs. *asn1(**A6E**)::GFP* (*asn2*Δ) Clone **1**					0.0043	**	Yes
*ASN1(**WT**)::GFP* (*asn2*Δ) Clone **1** vs. *asn1(**A6E**)::GFP* (*asn2*Δ) Clone **2**					0.0050	**	Yes
*ASN1(**WT**)::GFP* (*asn2*Δ) Clone **2** vs. *asn1(**A6E**)::GFP* (*asn2*Δ) Clone **1**					0.0049	**	Yes
*ASN1(**WT**)::GFP* (*asn2*Δ) Clone **2** vs. *asn1(**A6E**)::GFP* (*asn2*Δ) Clone **2**					0.0038	**	Yes
*asn1(**A6E**)::GFP* (*asn2*Δ) Clone **1** vs. *asn1(**A6E**)::GFP* (*asn2*Δ) Clone **2**					0.0237	*	No

Notes: ns (*p*-value > 0.05), * (*p*-value ≤ 0.05), and ** (*p*-value ≤ 0.01).

## Data Availability

The data presented in this study are available within the manuscript and its Supplementary Materials.

## Supplementary Materials

The following are available online at https://www.mdpi.com/article/10.3390/biology10040294/s1. Figure S1: Amino acid sequence alignment of human and yeast asparagine synthetases. Figure S2: Expression levels of Asn1p(WT)-GFP vs. Asn1p(A6E)-GFP (in *asn2*Δ background). Table S1: List of primers used for recombinant plasmid modification (by PCR-based, site-directed mutagenesis), making a DNA cassette for yeast transformation and preparing PCR products (of isolated yeast genomic DNA) for strain verification by DNA sequencing. Table S2: Numerical and statistical data of two yeast strains, *ASN1(WT)::GFP* (*asn2*Δ) and *asn1(A6E)::GFP* (*asn2*Δ), used to plot graphs of assembly kinetics, sodium azide treatment, and fresh glucose addition, as shown in Figure 4 and Figure 5.
