# Supplementary material for: Effects of A6E Mutation on Protein Expression and Supramolecular Assembly of Yeast Asparagine Synthetase
Source: Biology (Basel). 2021 Apr 3;10(4):294. doi: 10.3390/biology10040294 (PMC8065433; doi:10.3390/biology10040294)
Supplement: Supplementary file 1 [file biology-10-00294-s001.pdf]

```
P08243 ASNS_HUMAN           1   MCGIWALFGSDDCLSVQC---LSAMKIAHRGPDAFRFENVNGYTNCCFGFHRLAVVDPLF
P49089 ASNS1_YEAST          1   MCGIFAAFRHEDVHRYKPKALQLSKRIRHRGPDWSGNA---IKNSTIFVHERLAIVGVES
                                ****:* *  :*    :      : :* *****          ..  * ..***:*.

P08243 ASNS_HUMAN          58   GMQPIRVKKYPYLWLCYNGEIYNHKKMQQH-FEFEYQTKVDGEIILHLYDKGGIEQTICM
P49089 ASNS1_YEAST         58   GAQPITSSDGEY-MLCVNGEIYNHIQLREECADYEFGTLSDCEPIIPMYLKHDIDAP-KY
                                * ***  ..  *  ** ******* ::::.  ::*: *  * * *: :* * .*:

P08243 ASNS_HUMAN         117   LDGVFAFVLLDTANKKVFLGRDTYGVRPLFKAMTEDGFLAV--CSEAKGLVTLKHSATPF
P49089 ASNS1_YEAST        116   LDGMFAWTLYDAKQDRIVAARDPIGITTLYMGRSSASPKTVYFASELKCL------TDDC
                                ***:**:.* *: :.::. .**  *:  *: . :. .  :*  .** * *      :

P08243 ASNS_HUMAN         175   LKVEPFLPGHYEVLDLKPNGKVASVEMVKYHHCRDVPLHALYDNVEKLFPGFEIETVKNN
P49089 ASNS1_YEAST        170   DTITAFPPGHVYDSKTD--------KITRYFTPD------WL--DEKRIPSTPID--YMA
                                 .:  * ***    . .        ::.:*.              ** :*.  *:

P08243 ASNS_HUMAN         235   LRILFNNAVKKRLMTDRRIGCLLSGGLDSSLVAATLLKQLKEA-----------------
P49089 ASNS1_YEAST        212   IRHSLEKAVRKRLMAEVPYGVLLSGGLDSSLIASIAARETAKATNDVEPSTYDSKARHLA
                                :*  :::**:****::   * **********:*:   ::  :*

P08243 ASNS_HUMAN         278   ----------QVQYPLQTFAIGMEDSPDLLAARKVADHIGSEHYEVLFNSEEGIQALDEV
P49089 ASNS1_YEAST        272   GIDDDGKLHTAGWTSLHSFAIGLPNAPDLQAARKVAKFIGSIHHEHTFTLQEGLDALDDV
                                               *::****: ::*** ******..*** *:*  *. :**::***:*

P08243 ASNS_HUMAN         328   IFSLETYDITTVRASVGMYLISKYIRKNTDSVVIFSGEGSDELTQGYIYFHKAPSPEKAE
P49089 ASNS1_YEAST        332   IYHLETYDVTTIRASTPMFLLSRKIKAQG-VKMVLSGEGSDEIFGGYLYFAQAPSAAEFH
                                *: *****:**:***. *:*:*: *: :    :::*******:  **:** :***  : .

P08243 ASNS_HUMAN         388   EESERLLRELYLFDVLRADRTTAAHGLELRVPFLDHRFSSYYLSLPPEMRI--PK-NGIE
P49089 ASNS1_YEAST        391   TESVQRVKNLHLADCLRANKSTMAWGLEARVPFLDREFLQLCMNIDPNEKMIKPKEGRIE
                                 ** : :::*:* * ***:::* * *** ******:.* .  :.: *: ::  ** . **

P08243 ASNS_HUMAN         445   KHLLRETFED------SNLIPKEILWRPKEAFSDGITSVKNSWFKILQEYVEHQVDDAMM
P49089 ASNS1_YEAST        451   KYILRKAFDTTGEPDAKPYLPEEILWRQKEQFSDGVG---YSWIDGLKDTAEAVISDEMF
                                *::**::*:       .  :*:***** ** ****:     **:. *:: .*  :.* *:

P08243 ASNS_HUMAN         499   ANAAQKFPFNTPKTKEGYYYRQVFERHYPGRADW-LSHYWMP--KWINATDPSARTLTHY
P49089 ASNS1_YEAST        508   ASPKAEWGSDIPTTKEAFWYRLKFDALFPQKTVADTVMRWIPKADWGCAEDPSGRYAQIH
                                *.   ::  : *.***.::**  *:  :* ::       *:*  .*  * ***.*    :

P08243 ASNS_HUMAN         556   KSAVKA
P49089 ASNS1_YEAST        568   EKHIE-
                                :. ::
```

Figure S1. Amino acid sequence alignment of human and yeast asparagine synthetases. Amino acid sequences of human ASNS (P08243) and yeast Asn1p (P49089) were aligned using CLUSTALO available at www.uniprot.org. They showed 204 identical and 153 similar amino acid residues. The red box indicates the conserved alanine residue for our mutation study.

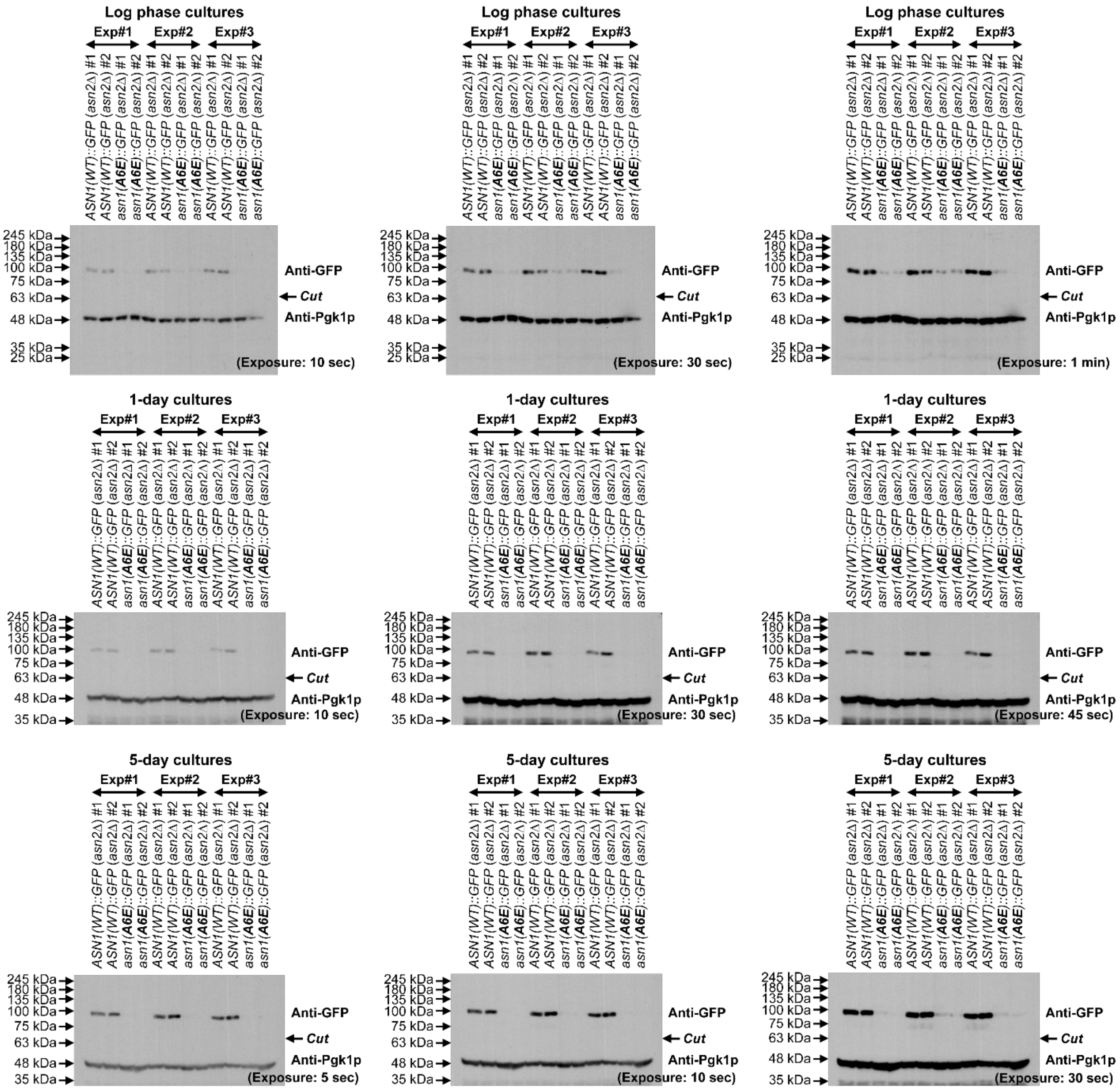

Figure s2. Expression levels of Asn1p(WT)-GFP vs. Asn1p(A6E)-GFP (in *asn2*Δ background). Two different clones of yeast *ASN1*(*WT*)*::GFP* (*asn2*Δ) and *asn1*(*A6E*)*::GFP* (*asn2*Δ) were grown in liquid YPD at 30 °C with shaking to log phase, saturation (1-day cultures), or stationary phase (5-day cultures). Then, 1, 5, or 10 OD_{600} cells for log phase, 1-day, and 5-day cultures, respectively, were taken to prepare whole cell extracts for SDS-PAGE and Western blot analysis. Each membrane was cut into 2 pieces between 75 and 63 kDa bands of the pre-stained protein ladder. The upper piece of each divided blot was used to detect GFP-tagged Asn1p with anti-GFP (91.7 kDa for Asn1p(WT)-GFP, and 91.8 kDa for Asn1p(A6E)-GFP). The lower piece of each divided blot was used to detect Pgk1p (as internal loading control) (44.7 kDa). Three independent experiments were performed to confirm the results.

Table S1. List of primers used for recombinant plasmid modification (by PCR-based, site-directed mutagenesis), to make a DNA cassette for yeast transformation, and to prepare PCR products (of isolated yeast genomic DNA) for strain verification by DNA sequencing.

Primer	Sequence	Description	Used with	Product size
**Primers for making DNA cassette** carrying (5′ to 3′): 50 bp upstream of the *ASN2* start codon, hygromycin resistance gene, and 50 bp downstream of the *ASN2* stop codon (**for transformation of yeast *BY4741***)<br>**Plasmid template:** pFA6a-hphMX6 (Addgene)<br>**Resulting yeast strain:** *asn2*Δ				
JW2193	5′- TTGCTTCCCAGTTAAGCTCACCAAAACACAAATACTCACTACAATACAATGGTCGACGGATCCCCGGG -3′	Forward; with 50 nt upstream of ***ASN2*** start codon and sequence homology to pFA6a-hphMX6 (underlined)	JW2194	1811 bp
JW2194	5′- CCGCATTTCTTGGTTCACTCGTCAATTATAAGAATACGATTGCGCTCGTAATCGATGAATTCGAGCTCG -3′	Reverse; with 50 nt downstream of ***ASN2*** stop codon and sequence homology to pFA6a-hphMX6 (underlined)	JW2193	
**Primers for verifying the success of deleting *ASN2* from yeast genome**<br>**DNA template:** genomic DNA isolated from selected yeast transformants<br>**Verified yeast strain:** *asn2*Δ				
JW2009	5′- CATTGACTCATGGCAAGATTTCTCC -3′	Forward; located 200 nt upstream of ***ASN2*** start codon	JW1986	1355 bp
JW1986	5′- CTCCATACAAGCCAACCACG -3′	Reverse; located at nt713-732 of hygromycin resistance coding sequence	JW2009	
**Mutagenic primers for introducing A6E mutation codon** (**GCC → GAA**) **to *ASN1* coding sequence** (**PCR-based, site-directed mutagenesis**)<br>**Plasmid template:** pFA6a-ASN1-GFP-kanMX6 (Noree et al., 2019)<br>**Resulting plasmid:** pFA6a-asn1(A6E)-GFP-kanMX6				
JW2295	5′- ATGTGTGGTATTTTCGAAGCTTTCAGGCACGAA -3′	Forward; A6E mutation codon (underlined)	JW2296	6593 bp
JW2296	5′- GTCGACCTGCAGCGTACGAAG -3′	Reverse	JW2295	
**Primers to prepare DNA cassette** carrying (5′ to 3′): 50 bp upstream of *ASN1* start codon + *asn1* coding sequence (with A6E mutation codon) + *GFP* + *kanR* + 50 bp downstream of *ASN1* stop codon (**for transformation of yeast *asn2*Δ**)<br>**Plasmid template:** pFA6a-asn1(A6E)-GFP-kanMX6<br>**Resulting yeast strain:** *asn1(A6E)::GFP* (*asn2*Δ)				
JW2301	5′- AAAAGTATAACTTGCTTTACGCTAAGGATATAAATCGGACGTAACTTAAG **ATG**TGTGGTATTTTCGAAGCTTTC -3′	Forward; with 50 nt upstream of ***ASN1*** start codon + the first 24 nt of ***ASN1*** coding sequence with A6E mutation codon (underlined)	JW2160	4240 bp
JW2160	5′- AAATATCTATAAGATTAATCCATAATTCTTTTTCTATTTTTTAATGTTATATCGATGAATTCGAGCTCG -3′	Reverse; with 50 nt downstream of ***ASN1*** stop codon and sequence homology to pFA6a-asn1(A6E)-GFP-kanMX6 (underlined)	JW2301	

Primer	Sequence	Description	Used with	Product size
**Primers used to prepare PCR products (using genomic DNA isolated from yeast transformants as DNA templates) for strain verification by DNA sequencing (Macrogen)**<br>**Verified yeast strain:** *asn1(A6E)::GFP* (*asn2*Δ)				
JW2010	5′- CTGCCCACTCGAGATGACAAATA -3′	Forward; located 200 nt upstream of ***ASN1*** start codon	JW1623	2829 bp
JW1623	5′- GCGACCTCATACTATACCTG -3′	Reverse; located 184 nt downstream of ***GFP*** coding sequence	JW2010	
**DNA sequencing primers**				
JW2010	5′- CTGCCCACTCGAGATGACAAATA -3′	Forward; located 200 nt upstream of ***ASN1*** start codon		
JW2038	5′- CGCATTCCTTCCACCCCAATAG -3′	Forward; located at nt601-622 of ***ASN1*** coding sequence		
JW2039	5′- ACATCGATCCAAATGAAAAGATG -3′	Forward; located at nt1301-1323 of ***ASN1*** coding sequence		
JW1623	5′- GCGACCTCATACTATACCTG -3′	Reverse; located 184 nt downstream of ***GFP*** coding sequence		

Table S2. Numerical and statistical data for two yeast strains, *ASN1(WT)::GFP* (*asn2*Δ) and *asn1(A6E)::GFP* (*asn2*Δ), used to plot graphs of assembly kinetics, sodium azide treatment, and fresh glucose addition, as shown in Figure 4 and 5.

**Assembly Kinetics**				
**Yeast strain**	**Clone #**	**% Cells with Asn1p-GFP structures (average ± SEM)**		
		**Log-phase**	**Day 1**	**Day 5**
*ASN1(**WT**)::GFP* (*asn2*Δ)	1	0.1 ± 0.13	26.1 ± 2.98	95.7 ± 0.93
	2	0.1 ± 0.11	33.7 ± 2.61	99.2 ± 0.79
asn1(***A6E***)::*GFP* (*asn2*Δ)	1	0.0 ± 0.00	13.4 ± 0.84	10.9 ± 1.39
	2	0.0 ± 0.00	10.7 ± 0.77	10.5 ± 1.36
P-value (**A6E** vs. **WT**)		0.0529^{ns} (two-tailed)	0.1788^{ns} (two-tailed)	0.0142* (two-tailed)

**Log-Phase Culture with Sodium Azide Treatment (15 min)**					
**Yeast strain**	**Clone #**	**% Cells with Asn1p-GFP structures (average ± SEM)**		**P-value (two-tailed)**	**Significantly different (< 0.05)?**
		**(1: 100) sterile water: log-phase culture**	**(1: 100) 1M NaN_3: log-phase culture**		
*ASN1(**WT**)::GFP* (*asn2*Δ)	1	0.1 ± 0.09	71.3 ± 8.84	0.0153*	Yes
	2	0.0 ± 0.00	57.6 ± 12.12	0.0415*	Yes
asn1(***A6E***)::*GFP* (*asn2*Δ)	1	0.0 ± 0.00	1.6 ± 0.81	0.1883^{ns}	No
	2	0.0 ± 0.00	2.7 ± 1.44	0.2060^{ns}	No

**5-Day Culture with Fresh Glucose Treatment (15 min)**					
**Yeast strain**	**Clone #**	**% Cells with Asn1p-GFP structures (average ± SEM)**		**P-value (two-tailed)**	**Significantly different (< 0.05)?**
		**(1: 20) sterile water: 5-day culture**	**(1: 20) 40% (*w/v*) glucose: 5-day culture**		
*ASN1(**WT**)::GFP* (*asn2*Δ)	1	96.6 ± 1.22	0.7 ± 0.39	0.0002***	Yes
	2	97.0 ± 0.26	0.8 ± 0.31	<0.0001****	Yes
asn1(***A6E***)::*GFP* (*asn2*Δ)	1	13.4 ± 2.02	14.9 ± 3.06	0.2873^{ns}	No
	2	11.8 ± 1.46	10.2 ± 1.15	0.5081^{ns}	No

Notes: ns (*p*-value > 0.05), * (*p*-value ≤ 0.05), ** (*p*-value ≤ 0.01), *** (*p*-value ≤ 0.001), and **** (*p*-value ≤ 0.0001).
